# 3,3′-Diindolylmethane improves antitumor immune responses of PD-1 blockade via inhibiting myeloid-derived suppressor cells

**DOI:** 10.1186/s13020-022-00638-z

**Published:** 2022-06-30

**Authors:** Qi Sun, Lin Xiao, Zhiying Cui, Yaping Yang, Junting Ma, Zhen Huang, Junfeng Zhang, Jiangning Chen

**Affiliations:** 1 State Key Laboratory of Analytical Chemistry for Life Sciences and State Key Laboratory of Pharmaceutical Biotechnology, School of Life Sciences, Nanjing University, 210023 Nanjing, China; 2 Department of Pharmacology, School of Basic Medical Sciences, Anhui Medical University, 230032 Hefei, China

**Keywords:** 3,3′-Diindolylmethane, Cancer immunotherapy, Adjunctive therapy, PD-1 blockade, Myeloid-derived suppressor cells

## Abstract

**Background:**

Immune checkpoint inhibitors that target programmed cell death protein 1 (PD-1) have obtained encouraging results, but a fraction of tumor patients failed to respond to anti-PD-1 treatment due to the existence of multiple immune suppressive elements such as myeloid-derived suppressor cells (MDSCs). Traditional Chinese medicine or natural products from medicinal plants could enhance immunity and may be helpful for cancer immunotherapy. As a digestive metabolite from cruciferous plants, 3,3′-diindolylmethane (DIM) has been widely used in chemotherapy, but its influence on cancer immunotherapy remains unclear. Here we investigate the function of DIM on MDSCs and examine the therapeutic effects of DIM in conjunction with PD-1 antibody against mouse tumors.

**Methods:**

Flow cytometry analysis, Western blot analysis and qRT-PCR assay were used to examine the inhibitory effects and mechanisms of DIM on MDSCs in vitro and in vivo. The therapeutic effects of DIM on cancer immunotherapy by PD-1 antibody were evaluated in mouse models of breast cancer and melanoma tumor.

**Results:**

DIM exerted the inhibitory effect on MDSCs via downregulating miR-21 level and subsequently activating PTEN/PIAS3-STAT3 pathways. Adoptive transfer of MDSCs impaired the therapeutic effects of DIM, indicating that the antitumor activity of DIM might be due to the suppression of MDSCs. Furthermore, in mouse models of breast cancer and melanoma tumor, the addition of DIM can enhance the therapeutic effect of PD-1 antibody through promoting T cells responses, and thereby inhibiting tumor growth.

**Conclusions:**

Overall, the strategy based on the combination treatment of anti-PD-1 antibody and DIM may provide a new approach for cancer immunotherapy. Cruciferae plants-rich diet which contains high amount of DIM precursor may be beneficial for cancer patients that undergo the anti-PD-1 treatment.

**Supplementary Information:**

The online version contains supplementary material available at 10.1186/s13020-022-00638-z.

## Background

Recently, cancer immunotherapeutic strategies based on blocking the immune checkpoint of programmed cell death protein-1 (PD-1) lead to the successful reinvigoration of T cell function and have shown favor therapeutic effects in multiple cancer types [1, 2]. However, increasing evidence shows that myeloid-derived suppressor cells (MDSCs), regulatory T cells, tumor-associated macrophages, etc., are involved in tumor-mediated immunosuppressive responses, which are the major cause of poor outcome in cancer patients treated by the immune checkpoint blockers [3–6]. Among the above immunosuppressive cells in the tumor microenvironment, MDSCs are one of the main drivers in forming the immunosuppressive environment [7]. MDSCs exhibit extraordinary potential in promoting T cell dysfunction via multiple mechanisms such as arginase 1 (Arg1), inducible NO synthase (iNOS) and indoleamine 2,3-dioxygenase [8]. Both clinical data and animal model results indicate that the effect of cancer immunotherapy is inversely related to the MDSCs population [6, 9]. Therefore, a promising strategy of cancer immunotherapy requires not only an immune checkpoint blockade (i.e. increase of immune activation) but also a reduction of suppressive elements, such as MDSCs in the immune system [10–12].

Traditional Chinese medicine or natural products from medicinal plants have been widely used in cancer therapy via multiple ways, in which they directly inhibit tumor growth, reduce side effects from other anticancer therapies and activate antitumor immunity [13–15]. Particularly, the immunomodulatory function of traditional Chinese medicine have been paid much attention [16]. For example, Chinese medicine compounds (Yanghe Decoction, Baoyuan Jiedu decoction and Ze-Qi-Tang) can inhibit the function of MDSCs and abate the tumor-mediated immunosuppression, finally lead to the enhance of antitumor immune responses [17–19]. Thus, seeking optimized Chinese medicine derived biological components targeting MDSCs with high efficiency and low toxicity may lead to the enhanced therapeutic effect of PD-1 blockers and improve the clinical outcome of immunotherapy.

3,3′-Diindolylmethane (DIM) is a natural component formed during the autolytic breakdown of indole-3-carbinol presented in cruciferous plants and exert anti-cancer activity via restraining proliferation, metastasis and promoting apoptosis of tumor cells [20, 21]. Therefore, cruciferous plants have promising anti-cancer potential, which may result from the high contents of DIM precursor (glucosinolates). Recent studies also demonstrated that DIM could protect against murine colitis models through affecting the functions of immune cells [22, 23]. However, the influence of DIM on MDSCs remains unclear in the tumor microenvironment. In this study, we first investigated the effect and the underlying mechanism of DIM on MDSCs and found that DIM could suppress the expansion and immune suppressive function of MDSCs via suppressing miR-21 level and the following STAT3 activation. More importantly, we found that DIM treatment enhanced the antitumor immune responses of anti-PD-1 in tumor-bearing mouse models. These findings might provide not only new insights into the function of DIM in the impairment of cancer immunosuppression but also clues for the establishment of traditional Chinese medicine therapy strategy of cancer immunotherapy.

## Materials and methods

### Reagents

DIM (purity > 98%), dimethylsulfoxide (DMSO), 2-hydroxypropyl-β-cyclodextrin and Con A were purchased from Sigma-Aldrich (St. Louis, MO, USA). Mouse GM-CSF and IL-6 were purchased from PeproTech (Rocky Hill, NJ, USA). MiR-21 precursors (pre-miR-21), miR-21 inhibitors (anti-miR-21) and the corresponding controls (pre-scramble or anti-scramble) were purchased from Gene Pharma (Shanghai, China). All primers used in the experiments were synthesized by Real Gene (Nanjing, China) and were shown in Additional file 2: Table S1. Mouse PD-1 antibody was obtained from Bioxcell (New Hampshire, USA). Fluorescence labeled antibodies used for flow cytometry were purchased from BioLegend (San Diego, CA, USA). Information of the antibodies used for Western blot in this study is listed in Additional file 2: Table S2. DIM was dissolved in DMSO for cell treatments. Due to the poor water solubility of DIM, it was formulated in 2-hydroxypropyl-β-cyclodextrin to increase its water solubility and to avoid the clearance of the mononuclear phagocytic system when used in vivo [24]. Briefly, 20 mg DIM was dissolved in 1 mL of 2-hydroxypropyl-β-cyclodextrin solution (molar ratio 1:10). The stock solution (20 mg/ml) were serially diluted in sterile PBS on the day of use to get certain concentrations according to our previous study [23].

### Animals and cell lines

Wild-type (WT) C57BL/6J and BALB/c mice were purchased from the Laboratory Animal Center of Nanjing University (Nanjing, China). *MiR-21*^loxp/loxp^ and *Cmv-Cre* mice in the C57BL/6 background were purchased from Biomodel Organism (Shanghai, China). *MiR-21*^loxp/loxp^ mice crossed with *Cmv-Cre* mice to generate *miR-21*^−/−^ mice. Then, they backcrossed to WT BABL/c mice for 7 generations to obtain *miR-21*^−/−^ mice with BALB/c background. *miR-21*^−/−^ mice were determined via PCR assay of tail genomic DNA. The primers used to distinguish WT and miR-21 knockout mice were shown in Additional file 2: Table S1. PCR procedures were as follows: 94 °C for 5 min, 94 °C for 30 s, 60 °C for 45 s and 72 °C for 45 s, for 30 cycles. Agarose gel electrophoresis was performed to examine the length of PCR products: WT (758 bp length) and miR-21 deficiency (480 bp length).

Experimental mice were housed at a specific pathogen free (SPF) environment under a ventilated, temperature-controlled room (23 °C) with a 12 h light/12 h dark cycle. All animal procedures were performed in accordance with the Guidelines for Care and Use of Laboratory Animals of Nanjing University and approved by the Animal Ethics Committee of Nanjing University.

According to the manufacturer’s instructions, we used cell isolation kit (Miltenyi Biotec, Bergisch Gladbach, Germany) to obtain primary MDSCs from bone marrow (BM) and spleen. The primary CD4^+^ and CD8^+^ T cells from the spleen were isolated using BD IMag Anti-Mouse CD4 Magnetic Particles-DM and BD IMag Anti-Mouse CD8 Particles-DM (BD Biosciences, San Jose, CA, USA). The murine breast cancer 4T1 cells (ATCC, Manassas, Virginia, USA) and primary T cells were cultured in RPMI 1640 containing 10% FBS (Life Technologies, Grand Island, NY, USA). The murine melanoma B16-F10 cells (ATCC) were cultured in DMEM containing 10% FBS. The cells were incubated at 37 °C with 5% CO_2_.

### Cell treatment

To induce BM-derived MDSCs, BM cells from WT mice (female, 5 weeks old) and *miR-21*^−/−^ mice (female, 5 weeks old) were stimulated with 40 ng/ml GM-CSF and 40 ng/ml IL-6 for 4 days in RPMI 1640 containing 10% FBS. Under some circumstances, different concentrations of DIM (10, 20, 40 or 80 µM) were added. Pre-miR-21, anti-miR-21, PIAS3 or PTEN expressing plasmid (VectorBuilder Inc., Guangzhou, China) and their corresponding controls were transfected into different treated MDSCs. Then part of the MDSCs was harvested to examine the expansion states by flow cytometry, and others were used to detect the miR-21 level, the mRNA levels of Arg1 and iNOS via qRT-PCR or the expression levels of potential miR-21 targeting genes by Western blot. For T cell proliferation assay, the CFSE-labeled CD4^+^ and CD8^+^ T cells were stimulated with 2 µg/ml Con A and cocultured with differently treated MDSCs at the ratio of 1:0.5 or 1:1 in 48-well plates for 3 days. Then T cell proliferation was analyzed by flow cytometry.

### Animal treatment

A total of 1 × 10^6^ 4T1 cells were injected into the fat pad of a mammary gland of WT BALB/c or *miR-21*^−/−^ mice with BALB/c background (female, 6–8 weeks old, 18–22 g) to establish mouse breast cancer model and a total of 1 × 10^6^ B16-F10 cells were injected subcutaneously into the left armpit of C57BL/6 mice (female, 6–8 weeks old, 18–22 g) to construct mouse melanoma cancer model.

Tumor-bearing mice were randomly assigned one week after tumor implantation and each group contained 8 mice. 4T1 Tumor-bearing mice were intraperitoneally injected with various dose of DIM (2, 5, 10 mg/kg DIM) at a volume of 100 µl three times a week from day 8 after tumor cell inoculation. Control mice were received with the same volume of 2-hydroxypropyl-β-cyclodextrin solution. To investigate the inhibitory effect of DIM on tumor growth through reducing MDSCs, DIM-treated 4T1 tumor-bearing mice were intravenously injected with 5 × 10^6^ of purified BM-MDSCs on day 13, 16, 19 after tumor cell inoculation. To examine whether DIM could improve the anti-tumor effect of anti-PD-1 mAb, breast cancer and melanoma tumor-bearing mice were intraperitoneally administrated with 10 mg/kg DIM three times a week from day 8 after tumor cell inoculation and intraperitoneally injected with anti-PD-1 mAb (0.25 mg/mouse) on day 11, 13, 15, 17, 19 after tumor cell inoculation. We measured the tumor size with a caliper every three days. On day 21, the mice were anaesthetized with pentobarbital sodium (80 mg/kg, i.p.) and blood samples were collected on ethylenediaminetetraacetic acid (EDTA) by cardiac puncture. Finally, the animals were killed by cervical dislocation. MDSCs from bone marrow, blood, spleen and tumor as well as T cells from blood and spleen were harvested for flow cytometry. Besides, IFN-γ level in the tumors from differently treated mice was measured by ELISA (eBioscience, San Diego, CA, USA).

### Flow cytometry analysis

The collected cells were blocked in 100 µl 1% bovine serum albumin for 30 min on ice and then stained with FITC-Gr-1, APC-CD11b, FITC-Ly-6 C, PE-Ly-6G, FITC-CD4, or APC-CD8 for another 30 min on ice. Flow cytometry was performed on a FACSCalibur device (Becton Dickinson, Franklin Lakes, NJ, USA) and the results were analyzed via using FCS Express V3 (DeNovo Software, Los Angeles, CA, USA).

### qRT-PCR assay

According to the manufacturer’s protocol, total RNA was isolated from cells by the Trizol reagent. For mRNA detection, 500 ng of total RNA were used for complementary DNA synthesis (One Step SYBR PrimeScript™ RT-PCR Kit, Takara, Shiga, Japan), according to the manufacturer’ instructions. For miR-21 detection, 2 µg of total RNA was used for first-strand DNA synthesis using AMV reverse transcriptase (Takara) and a stem-loop RT-primer (Life Technology). Real-time q-PCR was performed by 7300 real time PCR System (Applied Bio-systems, Foster City, CA) according to the manufacturer’s protocol. mRNA level was normalized to β-actin, while miR-21 expression was normalized to small nuclear RNA U6.

### Western blot

Total protein of cells was extracted with lysis buffer. Then the protein concentration was measured via Protein Quantitative Analysis Kit (shenergy Bio-color, Shanghai). 60 µg protein lysate was separated by 10% sodium dodecyl sulfate-polyacrylamide gel electrophoresis (SDS-PAGE), then transferred onto polyvinylidene difluoride (PVDF) membrane (Immobilon P, Millipore, Milford, MA). Membranes were blocked for 1 h using 5% skim milk and incubated with diluted primary antibody at 4 °C gently shaking overnight. Then the membrane was washed five times using PBST and incubated for 1 h at room temperature with secondary HRP-conjugated Antibody. The bound antibody was detected by ECL (Cell Signal Technology).

### Statistical analysis

Results are expressed as the mean ± standard error (SEM). Data was statistically analyzed using GraphPad Prism 8.0.2 (GraphPad Software Inc. La Jolla, CA, USA) and assessed for normality or homogeneity of variance. Significant differences between two groups were evaluated using two-tailed Student’s *t*-test. Differences between multiple groups were compared using one-way ANOVA with Dunnett’s tests or, if appropriate, using one-way ANOVA with post-hoc Bonferroni correction. The difference was considered significant when *p* < 0.05; ns = not significant.

## Results

### DIM suppressed the expansion and immunosuppressive function of BM-MDSCs

**Fig. 1:**
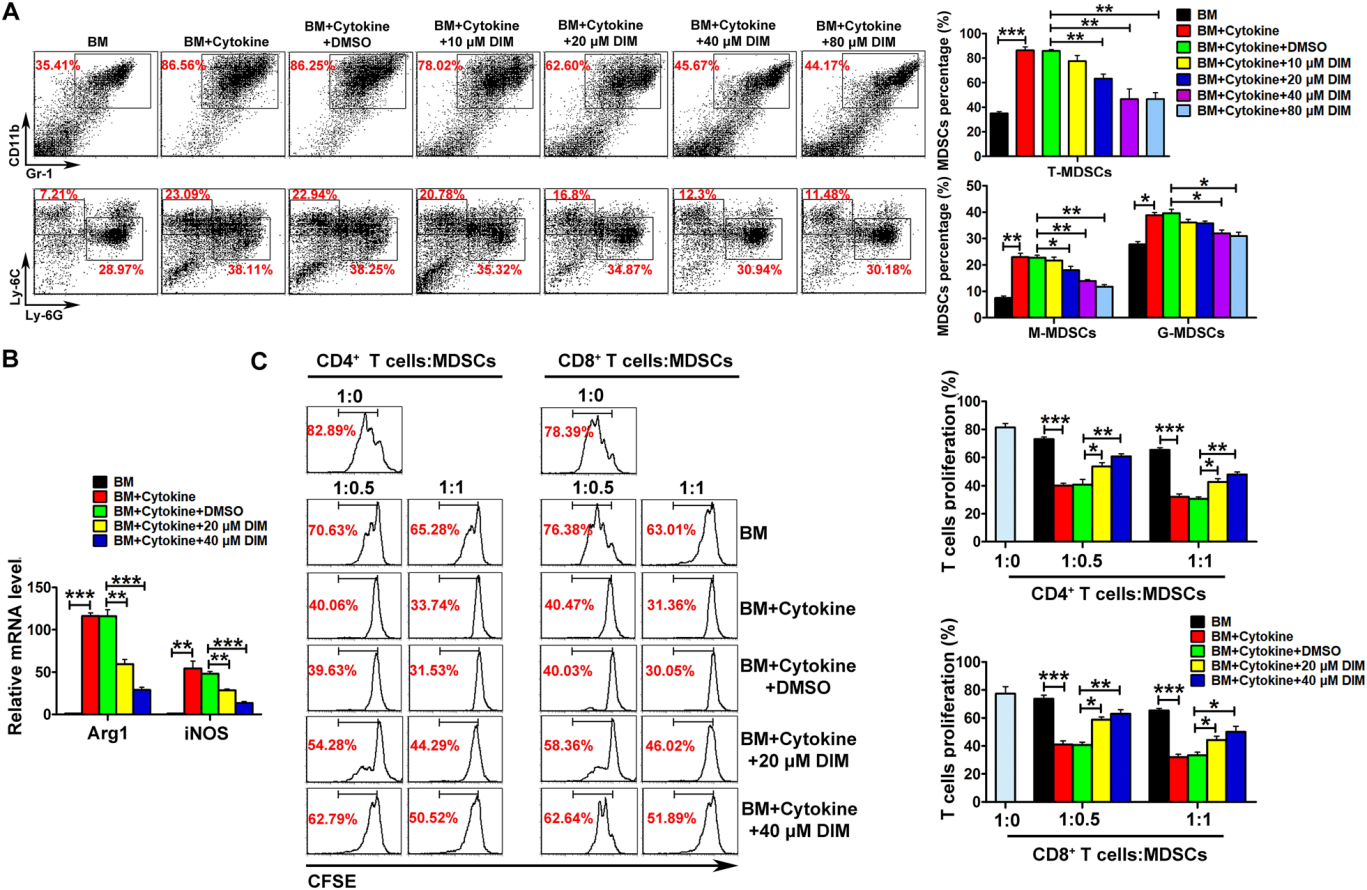
DIM inhibited the expansion and function of MDSCs in vitro.** A** BM-MDSCs were co-treated with 40 ng/ml GM-CSF and 40 ng/ml IL-6 and different doses of DIM (10 µM, 20 µM, 40 µM, 80 µM) for 4 days, and then analyzed by flow cytometry to examine the ratio of Gr-1^+^CD11b^+^ (total MDSCs, T-MDSCs), Ly-6G^+^CD11b^+^ (monocytic MDSCs, M-MDSCs) and Ly-6 C^+^CD11b^+^ cells (granulocytic MDSCs, G-MDSCs). **B** Relative mRNA levels of Arg1 and iNOS were determined in the BM-induced MDSCs treated with different concentrations of DIM. **C** CFSE-labeled CD4^+^ or CD8^+^ T cells were co-cultured with the BM-induced MDSCs at 1:0.5 and 1:1 ratio for 3 days, and then the T cell proliferation rate was assayed by flow cytometry. Data are representative results from three independent experiments and the results are expressed as the mean ± SEM. *p < 0.05, **p < 0.01 and ***p < 0.001

To evaluate the effect of DIM, we incubated BM-MDSCs with different concentrations of DIM. Results in Fig. 1A showed that DIM could suppress the induced ratio of BM-MDSCs in a concentration-dependent way. Further subsets analysis found that DIM could suppress the expansion of both subsets, granulocytic myeloid-derived suppressor cells (G-MDSCs) and monocytic myeloid-derived suppressor cells (M-MDSCs) (Fig. 1A). In addition, as functional markers of MDSCs, the mRNA levels of Arg1 and iNOS were decreased in DIM-treated BM-MDSCs (Fig. 1B). Cell co-culture study showed that BM-MDSCs could effectively inhibit CD4^+^ and CD8^+^ T cell proliferation, whereas DIM pre-treatment could partially abrogate the inhibitory effect of BM-MDSCs on T cell proliferation (Fig. 1C).

### DIM inhibited 4T1 tumor growth via suppressing MDSCs

**Fig. 2:**
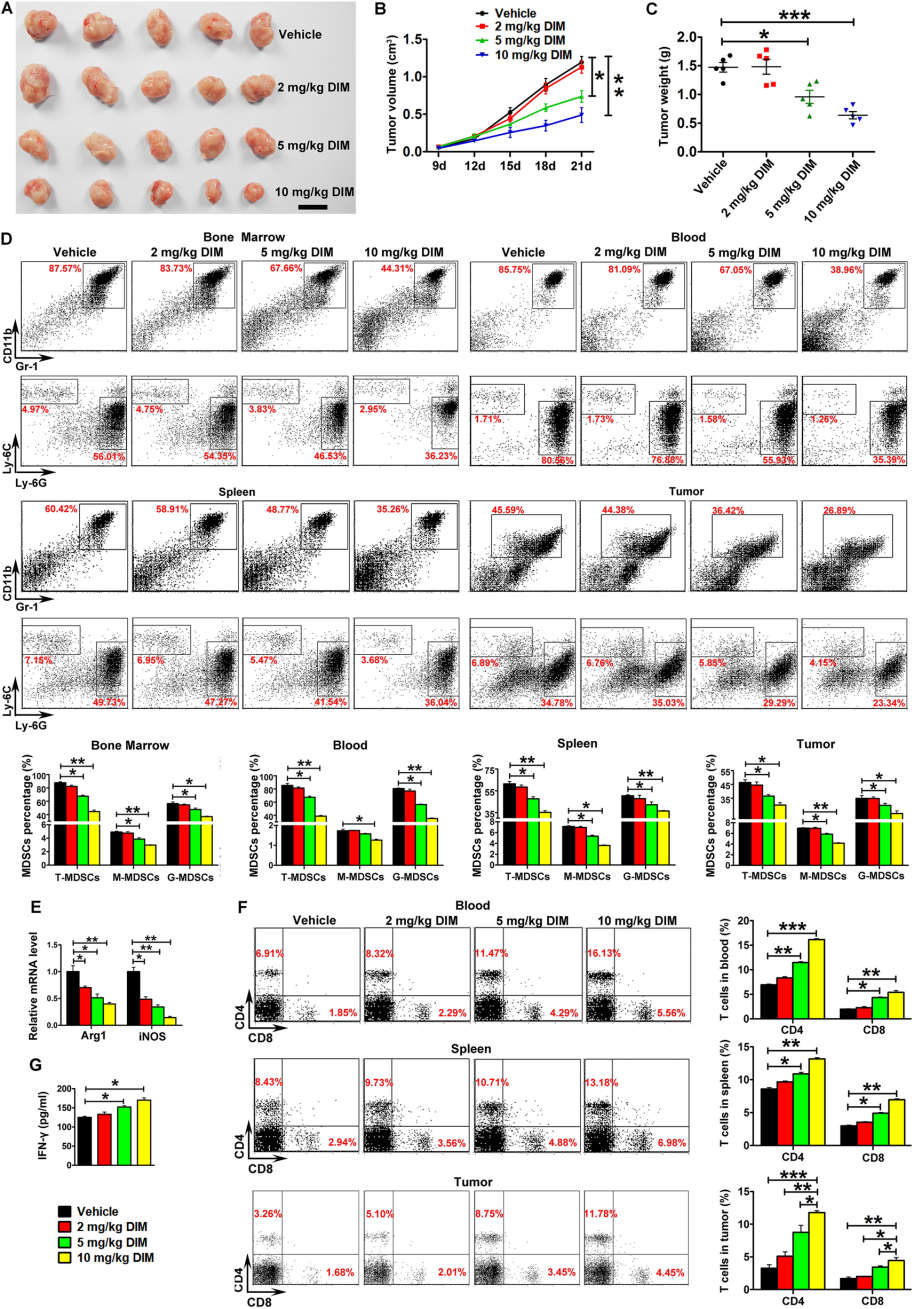
DIM treatment inhibited tumor growth in a dose-dependent manner via suppressing MDSCs. **A** Representative images of tumors, **B** tumor volume and **C** tumor weight were shown after 4T1 tumor-bearing mice were intraperitoneally injected with β-cyclodextrin or 2, 5, 10 mg/kg DIM three times a week for two weeks from day 8 after tumor cell inoculation (n = 8). **D** Representative flow cytometry data of total MDSCs and their subsets from bone marrow, blood, spleen and tumor were shown. **E** Relative mRNA levels of Arg1 and iNOS in MDSCs from spleen of 4T1 tumor-bearing mice treated with different doses of DIM. **F** The percentage of CD4^+^ and CD8^+^ T cells from blood, spleen and tumor in different doses DIM-treated tumor-bearing mice. **G** IFN-γ level in tumors was measured by ELISA. Data are representative results from three independent experiments and the results are expressed as the mean ± SEM. *p < 0.05, **p < 0.01 and ***p < 0.001. T-MDSC: total MDSCs; M-MDSC: monocytic MDSC; G-MDSC: granulocytic MDSC

WT mice bearing 4T1 tumors were treated with DIM three times a week for two weeks. The image of representative tumors collected at day 21 after implantation was shown in Fig. 2A. DIM treatment resulted in a marked inhibition of tumor growth (Fig. 2B) as well as the tumor weight (Fig. 2C) in a dose-dependent manner. Next, we tested the percentage of MDSCs from the bone marrow, blood, spleen and tumor. The results in Fig. 2D showed that DIM treatment reduced the percentages of total MDSCs (T-MDSCs), G-MDSCs and M-MDSCs from bone marrow, blood, spleen and tumor tissues in a dose-dependent manner. The mRNA levels of key effectors Arg1 and iNOS from splenic MDSCs were downregulated after DIM treatment (Fig. 2E), indicating that DIM may impair the immune suppressing effect of MDSCs. In addition, we found that 10 mg/kg DIM treatment could significantly elevate the percentages of CD4^+^ and CD8^+^ T cells in blood, spleen and tumor compared with the vehicle group (Fig. 2F). ELISA assay indicated the increase of IFN-γ level in tumor tissue from mice with DIM treatment (Fig. 2G).

### MDSCs adoptive transfer abolished the suppressing effect of DIM on tumor growth

**Fig. 3:**
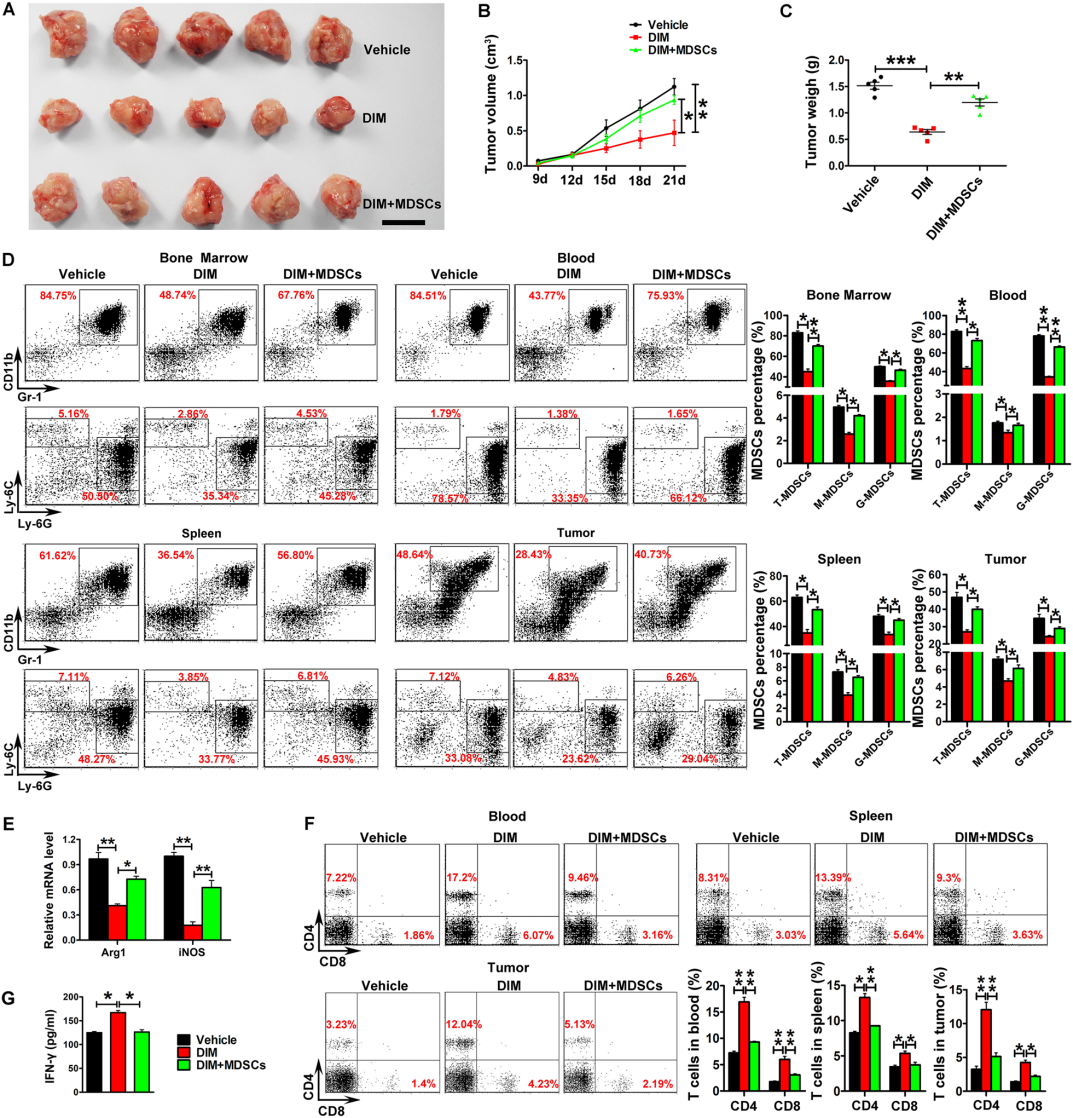
Adoptive transfer of MDSCs impaired antitumor effect of DIM in 4T1 tumor-bearing mice. **A** Representative images of tumors, **B** tumor volume and **C** tumor weight were shown after 4T1 tumor-bearing mice with the treatment of 10 mg/kg DIM (three times a week from day 8 after tumor cell inoculation) were intravenously (i.v.) injected with 5 × 10^6^ BM-derived MDSCs on day 13, 16, 19 after tumor cell inoculation (n = 8). **D** The percentage of total MDSCs and their subsets from bone marrow, blood, spleen and tumor was examined in DIM-treated mice after transferred with adoptive MDSCs. **E** Relative mRNA levels of Arg1 and iNOS in splenic MDSCs of 4T1 tumor-bearing mice co-treated with DIM and adoptive MDSC transfer. **F** The percentage of CD4^+^ and CD8^+^ T cells from blood, spleen and tumor was examined in DIM-treated mice after transferred with adoptive MDSCs. **G** IFN-γ level in tumor was measured by ELISA. Data are representative results from three independent experiments and the results are expressed as the mean ± SEM. *p < 0.05, **p < 0.01 and ***p < 0.001. T-MDSC: total MDSCs; M-MDSC: monocytic MDSC; G-MDSC: granulocytic MDSC

To further study the role of MDSCs in DIM-induced anti-tumor activity, BM-derived MDSCs were adoptively transferred into the 4T1 tumor-bearing mice via tail vein. We found that the adoptively transferred MDSCs could counteract the inhibition effect of DIM on 4T1 tumor growth (Fig. 3A–C). As shown in Fig. 3D, in contrast to vehicle-treated group, the ratio of total MDSCs as well as G-MDSCs and M-MDSCs decreased in the DIM-treated group, while MDSCs adoptive transfer could recover the percentage of MDSCs in the DIM-treated mice. Meanwhile, the mRNA levels of Arg1 and iNOS from splenic MDSCs were also increased after the replenishment of exogenous MDSC (Fig. 3E). Moreover, compared with the DIM-treated group, the percentage of CD4^+^ and CD8^+^ T cells was found to decrease in spleen, blood and tumor tissue from MDSCs adoptive transfer group (Fig. 3F). MDSCs adoptive transfer also led to the downregulated level of IFN-γ in tumor tissue (Fig. 3G).

### DIM could enhance antitumor immune responses of PD-1 antibody in tumor-bearing mouse models

**Fig. 4:**
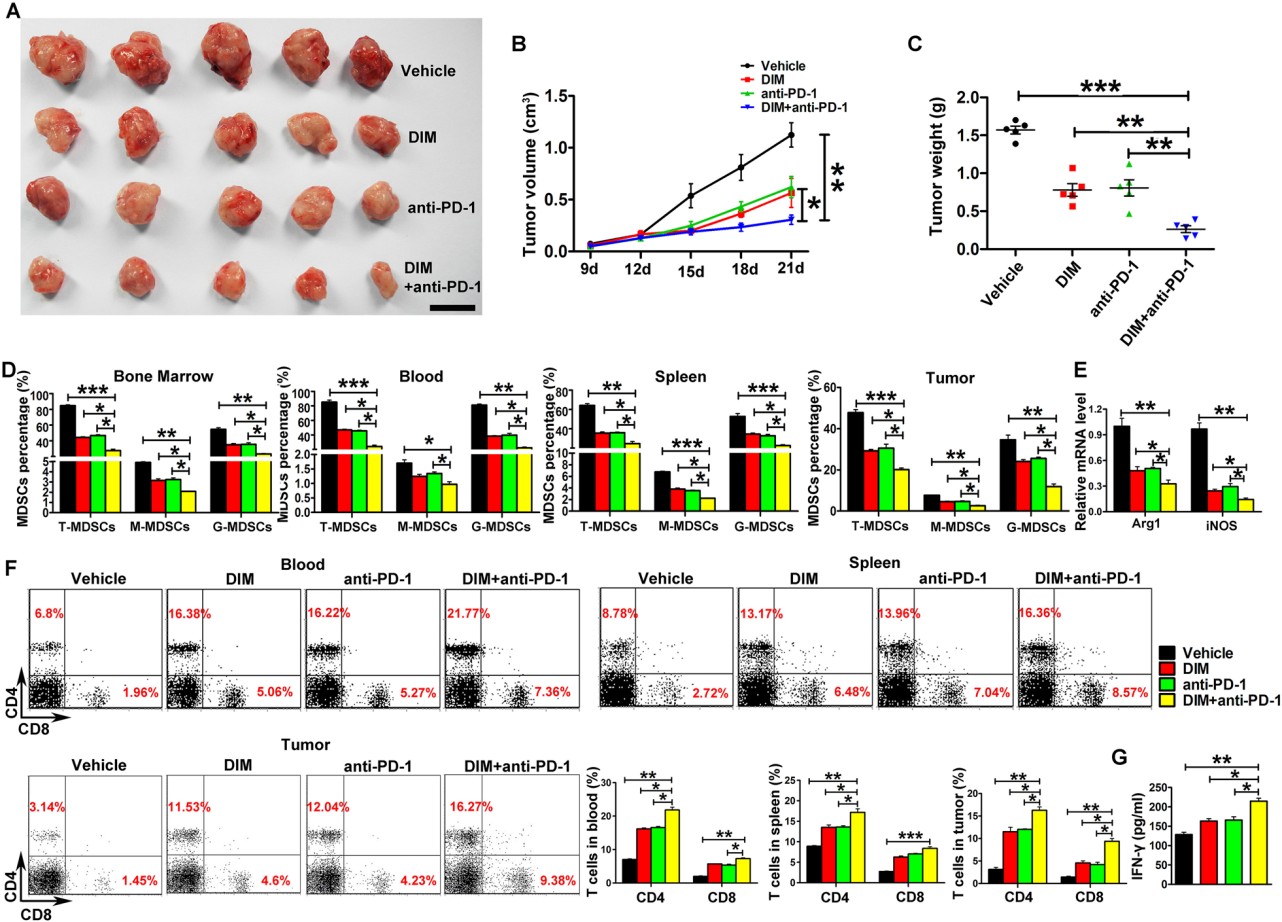
DIM treatment enhanced antitumor immune responses of anti-PD-1 in 4T1 tumor-bearing mice. **A** Representative images of tumors, **B** tumor volume and **C** tumor weight were shown after 4T1 tumor-bearing mice were treated with 10 mg/kg DIM three times a week for 2 weeks from day 8 after tumor cell inoculation and were intraperitoneally injected with anti-PD-1 mAb (0.25 mg/mouse) or isotype control antibody every two days from day 11 to 19 after tumor cell inoculation (n = 8). **D** The percentage of total MDSCs and their subsets from bone marrow, blood, spleen and tumor was analyzed by flow cytometry. **E** Relative mRNA levels of Arg1 and iNOS in MDSCs from spleen of 4T1 tumor-bearing mice after co-treated with DIM and anti-PD-1. **F** The percentage of CD4^+^ and CD8^+^ T cells from blood, spleen and tumor in 4T1 tumor-bearing mice after co-treated with DIM and anti-PD-1. **G** IFN-γ level in tumor was measured by ELISA. Data are representative results from three independent experiments and the results are expressed as the mean ± SEM. *p < 0.05, **p < 0.01 and ***p < 0.001. T-MDSC: total MDSCs; M-MDSC: monocytic MDSC; G-MDSC: granulocytic MDSC

The influence of DIM on the therapeutic effect of PD-1 antibody was evaluated in the mouse models of breast cancer (Fig. 4) and melanoma tumor (Additional file 1: Fig. S1). Anti-PD-1 treatment alone moderately reduced the tumor growth as well as DIM treatment. However, co-treatment with anti-PD-1 and DIM provided a more significant inhibition to tumor growth compared with the vehicle or the single treatment group (Fig. 4A–C and Additional file 1: Fig. S1 A–C). The ratio of MDSCs from bone marrow, blood, spleen and tumor tissue and the mRNA levels of Arg1 and iNOS from splenic MDSCs were found to decrease in the group co-treated with anti-PD-1 and DIM compared with the single treatment group (Fig. 4D, E and Additional file 1: Fig. S1D, E). Moreover, the combined treatment with anti-PD-1 and DIM could increase the percentage of CD4^+^ and CD8^+^ T cells in spleen, blood and tumor tissue compared with the single treatment group (Fig. 4F and Additional file 1: Fig. S1F). We also found that the combination treatment of PD-1 blockade and DIM induced a higher concentration of IFN-γ (Fig. 4G and Additional file 1: Fig. S1G).

### DIM inhibited immunosuppressive function of MDSCs via targeting miR-21

**Fig. 5:**
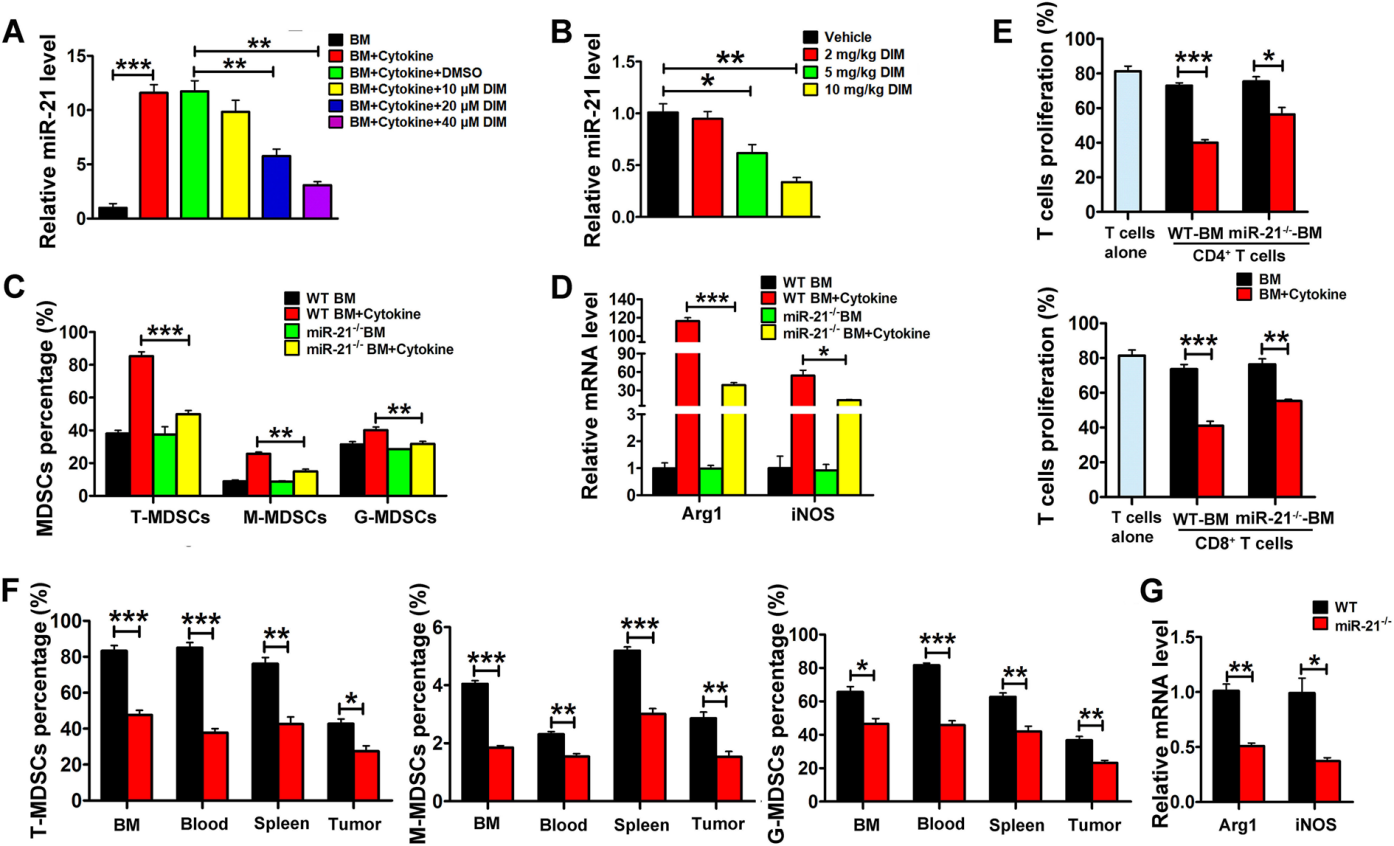
DIM treatment resulted in the reduction of miR-21 which inhibited the expansion and function of MDSC. **A** Relative miR-21 level in BM-induced MDSCs (in the condition of 40 ng/ml GM-CSF and 40 ng/ml IL-6 treated for 4 days) and **B** in the primary spleen MDSCs from 4T1 tumor-bearing mice after treated with DIM. **C** The percentage of total MDSCs and their subsets in BM-derived/induced MDSCs from WT mice and *miR-21*^−/−^ mice. **D** Relative mRNA levels of Arg1 and iNOS were monitored by qRT-PCR in BM- induced MDSCs from WT or miR-21^−/−^ mice. **E** BM cell or BM-induced MDSCs from WT or miR-21^−/−^ mice were cocultured with CFSE-labeled CD4^+^ and CD8^+^ T cells at a 1:2 ratio for 3 days, and T cell proliferation rate was evaluated by flow cytometry. **F** Primary MDSCs from bone marrow, blood, spleen and tumor were analyzed by flow cytometry in WT or miR-21^−/−^ 4T1 tumor-bearing mice. **G** Relative mRNA levels of Arg1 and iNOS in MDSCs from spleen of WT or *miR-21*^−/−^ 4T1 tumor-bearing mice. Data are representative results from three independent experiments and the results are expressed as the mean ± SEM. *p < 0.05, **p < 0.01 and ***p < 0.001. T-MDSC: total MDSCs; M-MDSC: monocytic MDSC; G-MDSC: granulocytic MDSC

Previous studies indicated that DIM could decrease miR-21 level in hepatic stellate cells during liver fibrosis [25] and miR-21 played a key role in promoting the expansion of MDSCs in lung cancer model [26]. We wondered whether DIM affected the expansion and immunosuppressive function of MDSCs through targeting miR-21. Our results showed that DIM treatment inhibited the level of miR-21 in BM-induced MDSCs and primary spleen MDSCs from 4T1 tumor-bearing mice in a dose-dependent manner (Fig. 5A, B). In this study, *miR-21*^−/−^ mice were conducted. As shown in Additional file 1: Fig. S2A, B, the mice were homozygous for miR-21 deletion and the loss of miR-21 in BM-MDSCs were confirmed in *miR-21*^−/−^ mice. The percentage of BM-induced MDSCs (including G-MDSCs and M-MDSCs subsets) and the mRNA levels of Arg1 and iNOS in BM-induced MDSCs from *miR-21*^*−/−*^ mice were much lower than that in WT mice (Fig. 5C, D). T cell proliferation assay demonstrated that the BM-induced MDSCs from *miR-21*^*−/−*^ mice showed the impaired suppressing effect on the proliferation of CD4^+^ and CD8^+^ T cells (Fig. 5E). We examined the ratio and phenotype of primary MDSCs in the bone marrow, blood, spleen, and tumor tissue from mice bearing 4T1 tumor. As shown in Fig. 5 F, the significantly decreased ratio of T-MDSCs, G-MDSCs and M-MDSCs was found in miR-21^−/−^ mice compared to WT mice. The mRNA levels of Arg1 and iNOS in splenic MDSCs from *miR-21*^*−/−*^ mice also decreased (Fig. 5G).

**Fig. 6:**
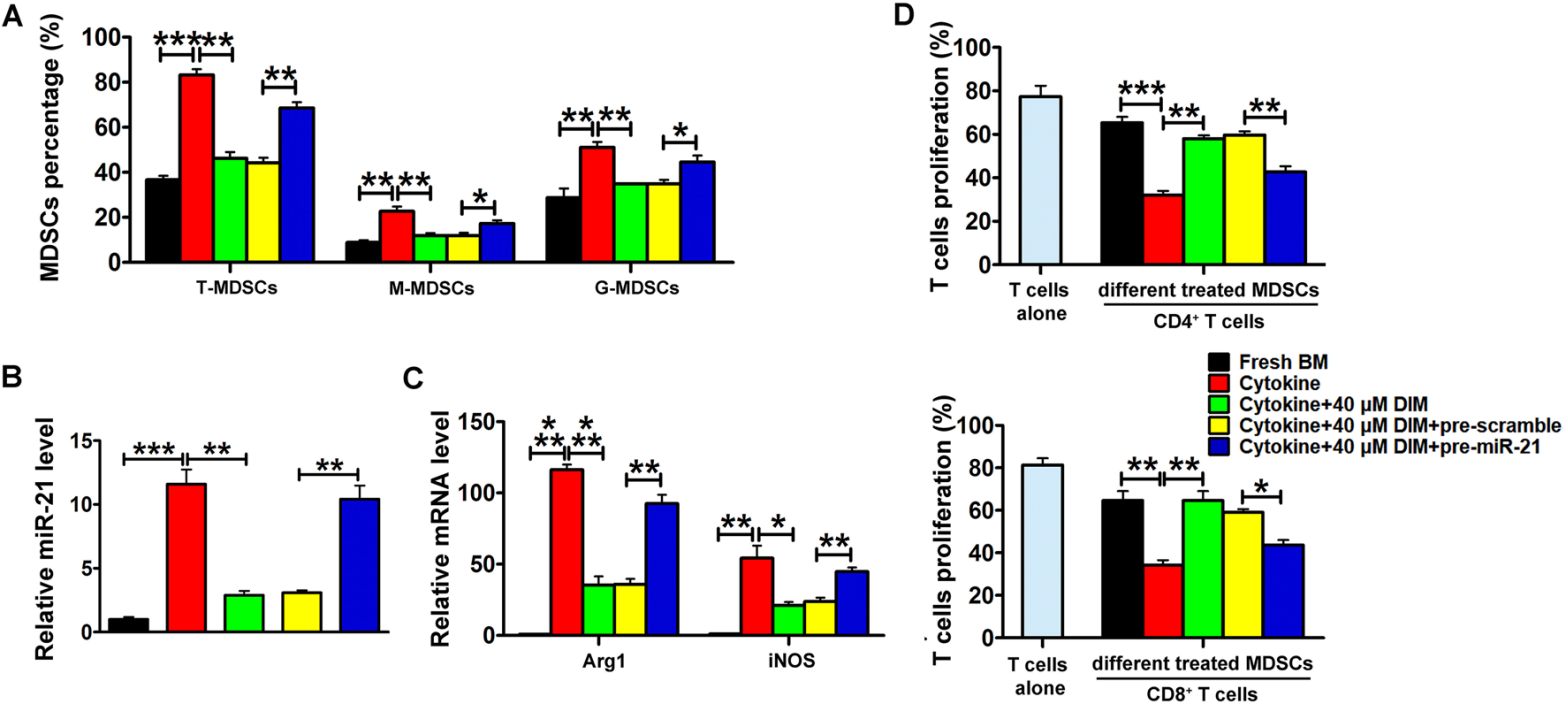
miR-21 recovery impaired the effects of DIM on MDSCs. **A** The percentage of total MDSCs and their subsets after BM-induced MDSCs (in the condition of 40 ng/ml GM-CSF and 40 ng/ml IL-6) were treated with DIM and miR-21 precursors for 4 days and examined by flow cytometry. **B** Relative miR-21 level and **C** Arg1/iNOS mRNA levels were examined in differently treated MDSCs. **D** The percentage of CD4^+^ and CD8^+^ T cells was measured by flow cytometry after BM-induced MDSCs were cocultured with DIM and pre-miR-21. Data are representative results from three independent experiments and the results are expressed as the mean ± SEM. *p < 0.05, **p < 0.01 and ***p < 0.001. T-MDSC: total MDSCs; M-MDSC: monocytic MDSC; G-MDSC: granulocytic MDSC

In addition, the miR-21 recovery experiment results showed that DIM inhibited the induced ratio of both total MDSCs and MDSCs subsets from BM cells, whereas overexpression of miR-21 rescued the induced ratio of total MDSCs, G-MDSCs and M-MDSCs (Fig. 6A). As shown in Fig. 6B, the level of miR-21 decreased in DIM-treated BM-MDSCs and pre-miR-21 treatment could recover the level of miR-21. Meanwhile, the mRNA levels of Arg1 and iNOS were markedly elevated in BM-MDSCs co-treated with pre-miR-21 and DIM (Fig. 6C). BM-MDSCs co-treated with pre-miR-21 and DIM strongly suppressed the proliferation of CD4^+^ and CD8^+^ T cell (Fig. 6D).

### DIM mediated miR-21 reduction inhibited MDSCs expansion via STAT3 signaling pathway

**Fig. 7:**
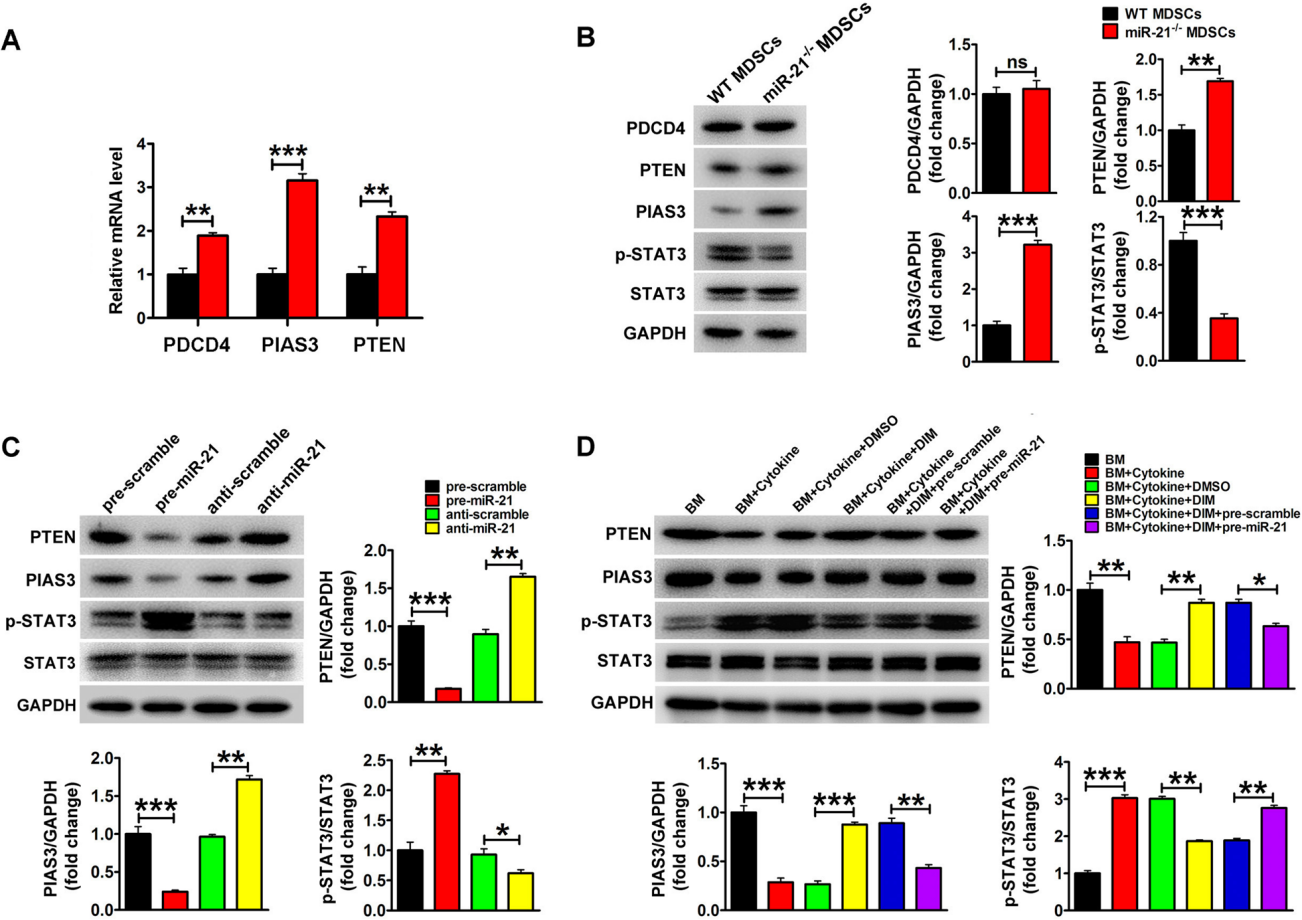
DIM mediated inhibitory effect on MDSCs depended on miR-21/STAT3 signaling pathway. **A** Relative mRNA levels and **B** protein levels of miR-21 targeting genes in the isolated BM cells from WT mice and *miR-21*^−/−^ mice after they were stimulated with 40 ng/ml GM-CSF and 40 ng/ml IL-6 for 4 days. **C** The protein levels of PTEN, PIAS3 and STAT3, p-STAT3 were examined in BM cells from WT mice and *miR-21*^−/−^ mice after they were stimulated and then transfected with miR-21 precursors, inhibitor and their matched controls. **D** The protein levels of PTEN, PIAS3 and STAT3, p-STAT3 were examined in BM-induced MDSCs after treated with DIM and miR-21 precursors for 4 days. Data are representative results from three independent experiments and the results are expressed as the mean ± SEM. *p < 0.05, **p < 0.01 and ***p < 0.001. T-MDSC: total MDSCs; M-MDSC: monocytic MDSC; G-MDSC: granulocytic MDSC

To identify the potential miR-21 targets related to the function of MDSCs, several reported miR-21 targeting genes (Spry1, Spry2, PDCD4, PIK3R1, Tipe2, btg2, PIAS3, PTEN, MEF2C and cyclinD1) were examined in BM-MDSCs from WT mice and *miR-21*^−/−^ mice. We found that the mRNA levels of PDCD4, PIAS3 and PTEN increased in BM-MDSCs from *miR-21*^−/−^ mice when compared with that from WT mice (Fig. 7A), whereas the mRNA levels of other genes were not changed (data not shown). Additionally, the protein levels of PTEN and PIAS3 were upregulated in the induced MDSCs of miR-21^−/−^ mice while the expression level of PDCD4 remained unchanged (Fig. 7B). Given the fact that both PTEN and PIAS3 are negative regulators of JAK2/STAT3 signaling pathway, we examined the phosphorylation levels of STAT3 in BM-MDSCs from WT mice and *miR-21*^−/−^ mice and found the decreased p-STAT3 levels in BM-MDSCs from *miR-21*^−/−^ mice (Fig. 7B). As shown in Fig. 7C, the increased miR-21 level in induced BM-MDSCs resulted in the reduction of PTEN and PIAS3 and the increased level of p-STAT3. In contrast, depleting miR-21 obtained the opposite result. Moreover, overexpressing either PIAS3 or PTEN could partly abrogated the effects of miR-21 on the phosphorylation of STAT3, which further indicated that miR-21 regulated STAT3 signaling pathway via targeting PIAS3 and PTEN (Additional file 1: Fig. S3). As shown in Fig. 7D, we found that miR-21 overexpression could decrease the protein levels of PTEN and PIAS3, and enhance the level of p-STAT3 compared with DIM alone treated MDSCs, which indicated DIM could affect MDSCs through miR-21/PTEN/STAT3 and miR-21/PIAS3/STAT3 pathways.

### MiR-21 deficiency attenuated the inhibitory effects of DIM on MDSCs expansion and tumor growth

To determine whether DIM mediated inhibitory effect on MDSCs via targeting miR-21/STAT3 signaling pathway, we also evaluated the therapeutic effects of DIM in *miR-21*^−/−^ mice bearing 4T1 tumor. As shown in Additional file 1: Fig. S4A–D, *miR-21*^−/−^ mice exhibited delayed tumor growth and lower MDCSs ratio compared with WT mice. In addition, *miR-21*^−/−^ tumor-bearing mice failed to respond to treatment with DIM. As shown in Additional file 1: Fig. S4E, F, DIM treatment could not affect the miR-21 level and protein levels of PTEN, PIAS3 and p-STAT3 in the splenic MDSCs from *miR-21*^−/−^ tumor-bearing mice. Additionally, DIM treatment showed neglectable influence on the mRNA levels of Arg1 and iNOS in splenic MDSCs from *miR-21*^−/−^ tumor-bearing mice (Additional file 1: Fig. S4G). We further found that DIM treatment could not improve the percentage of CD4 + and CD8 + T cells and the concentration of IFN-γ in *miR-21*^−/−^ tumor-bearing mice (Additional file 1: Fig. S4H, I).

## Discussion

The favor outcome has been achieved in a subset of magnificent cancer patients by the blockade of immune checkpoints, but the clinical benefits of PD-1 inhibition are not universal due to the suppressive microenvironment [27]. Thus, developing novel therapies in combination with PD-1 blockade to reverse immune tolerance would be necessary. Here, we demonstrated the inhibitory effect of DIM on the expansion and immunosuppressive function of MDSCs. Moreover, the combined treatment with the application of DIM and PD-1 blockade significantly increased the ratio of CD4^+^ and CD8^+^ T cells and enhanced IFN-γ secretion, ultimately suppressing tumor growth. Our findings indicate that DIM can be developed as a therapeutic reagent to enhance the antitumor immune responses of PD-1 blockade.

Cruciferous plants such as *Brassica alba* (L.) Boiss and *Brassica oleracea* var. *capitata* L. contain large amounts of glucosinolates and these chemicals are broken down to from biologically active molecules during digestion [28]. The potential anti-cancer effects of Cruciferous plants and their derived active components have been already reported [29–31]. As the most well-known breakdown products, DIM has been widely studied as an efficient chemoprevention against a range of cancers including melanoma, breast, prostate and colorectal cancers, mostly prompted by its ability to affect tumor cells, including anti-proliferation, apoptosis induction, etc. [32, 33]. Although previous studies have indicated that DIM exerts modulatory activity in different population of immune cells [23, 34], its effect on MDSCs remains unclear. In the current study, we observed that DIM treatment could decrease MDSC subsets both in vivo and in vitro. Moreover, adoptive transfer of MDSCs caused the impaired therapeutic effect of DIM in mouse 4T1 tumor models. Therefore, in addition to directly affect the tumor cells, DIM may exert its anti-tumor ability possibly through acting on MDSCs and disturbing the immune tolerance toward tumor.

MDSCs exhibit immune suppressing activity on different cell types of the immune system and T cell are the main targets of MDSCs. MDSCs consist of two major subtypes of cells termed G-MDSCs and M-MDSCs. M-MDSCs potently suppress antigen-specific and non-specific T cell responses via expressing high amounts of NO, Arg1 and immune suppressive cytokines. Meanwhile, G-MDSCs render T-cells unresponsive to antigen-specific stimulation through producing high levels of ROS [8, 35]. Moreover, MDSCs can disrupt the homing of T cells and induce the differentiation and infiltration of T regulatory cells. Cell co-culture studies further demonstrated that DIM-treated MDSCs failed to inhibit CD4^+^ and CD8^+^ T cell growth. As MDSCs are potent inhibitors of T lymphocytes proliferation and activity [36, 37] and the increased ratio of MDSCs shows correlation with the decreased efficacy of immunotherapies [10, 38], the increased T cell population in DIM-treated mice may be beneficial for maximizing the antitumor effects of PD-1 blockade. Meanwhile, PD-1 blockers also led to the inhibition of MDSCs expansion and function, which is consistent with previous studies [39, 40]. It is possible that PD-1 inhibition prevents the generation of MDSCs from myeloid progenitors and PD-1 blocker mediated T cell activation led to the apoptosis of MDSCs. More than expected, the combined application of PD-1 antibody and DIM exhibited the significantly enhanced antitumor effect compared with using individual reagent. This is an important effect to bring to the clinic, highlighting that combination therapy with DIM and PD-1 blockade may be essential to completely regain T cell function.

Recent studies demonstrated that miRNAs may underlie the expansion of functional MDSCs [26, 41, 42]. Among these reported miRNAs, miR-21 was one of the most markedly upregulated miRNAs in MDSCs and could promote the induction of functional MDSCs [26]. In this study, miR-21 deletion in mice resulted in the decreased population of MDSCs, which further confirmed the key role of miR-21 in the induction and function of MDSC. Based on the rescue experiment and miR-21 deleted mice, we identified that PTEN and PIAS3 were the targets of miR-21 in MDSCs. As PTEN and PIAS3 has been proved to be the negative regulators of STAT3, an essential transcriptional factor during the MDSC’s life cycle, our experimental data further confirmed that miR-21 targeting PTEN and PIAS3, which play a synergistic role in suppressing STAT3 activation during the process of inducing functional MDSC [43, 44].

The present study indicated that DIM inhibited the expression of miR-21 in both induced BM-MDSCs and primary MDSCs from 4T1 tumor-bearing mice. Zhang et al. found that DIM down-regulated miR-21 expression in hepatic stellate cells during the liver fibrosis process [25]. However, the influence of DIM on miR-21 is not consistent with other reports. For example, DIM was reported to arrest the proliferation of breast cancer cell via increasing miR-21 level [45]. It is possible that different cell types and different physiological statuses may lead to distinct miR-21 level after DIM treatment. In this study, as recovering miR-21 abolished the suppressing effect of DIM on functional MDSCs in vitro and miR-21 deletion attenuated inhibitory effects of DIM on MDSCs function in vivo, we suggested that DIM mediated downregulation of miR-21 and inactivation of STAT3 could serve as the molecular mechanism underlying the inhibition of MDSCs by DIM.

In the present study, our findings demonstrated that DIM mediated miR-21 reduction inhibited MDSCs expansion via STAT3 signaling pathway. However, the current research also has limitations. First, this study indicated the therapeutic effects and underlying mechanism of DIM in tumor bearing mice. Whether Cruciferae plant itself can inhibit MDSCs function and improve the therapeutic effect remains unclear. Second, we demonstrated that DIM enhanced the therapeutic effect via inhibiting MDSCs function. We cannot exclude the possibility that DIM may affect other cell populations in the tumor microenvironment. Therefore, the following study will focus on the therapeutic effect of Cruciferae plant on tumor growth and the influence of DIM on various types of immune cells in the tumor microenvironment, which may be useful for setting up diet and the application of DIM as an adjuvant for cancer patients that undergo immunotherapy.

## Conclusions

Overall, DIM could potently reverse the suppressive tumor microenvironment through reducing the population and activity of MDSCs, which enhanced the antitumor immune responses of PD-1 blockade. Therefore, the adjunctive therapy of DIM may represent a promising strategy for cancer immunotherapy based on PD-1 blockade.

## Supplementary Information

**Additional file 1: Figures S1–4.**

**Additional file 2: Table S1.** The primer sequences used in this study. **Table S2.** The antibodies used in this study.

## Data Availability

The datasets used and/or analyzed during the current study available from the corresponding author on reasonable request.

## Abbreviations

Arg1: arginase 1
BM: Bone marrow
DIM: 3,3′-Diindolylmethane
G-MDSCs: Granulocytic myeloid-derived suppressor cells
iNOS: Inducible NO synthase
MDSCs: Myeloid-derived suppressor cells
M-MDSCs: Monocytic myeloid-derived suppressor cells
PD-1: Programmed cell death protein 1
PIAS3: Protein inhibitor of activated STAT3
PTEN: Phosphatase and tensin homolog
T-MDSCs: Total MDSCs
