# Supplementary material for: 3,3′-Diindolylmethane improves antitumor immune responses of PD-1 blockade via inhibiting myeloid-derived suppressor cells
Source: Chin Med. 2022 Jun 30;17:81. doi: 10.1186/s13020-022-00638-z (PMC9245307; doi:10.1186/s13020-022-00638-z)
Supplement: Supplementary file 1 — Additional file 1: Figures S1–4. [file 13020_2022_638_MOESM1_ESM.docx]

**Additional file 1: Figure S1-4.**


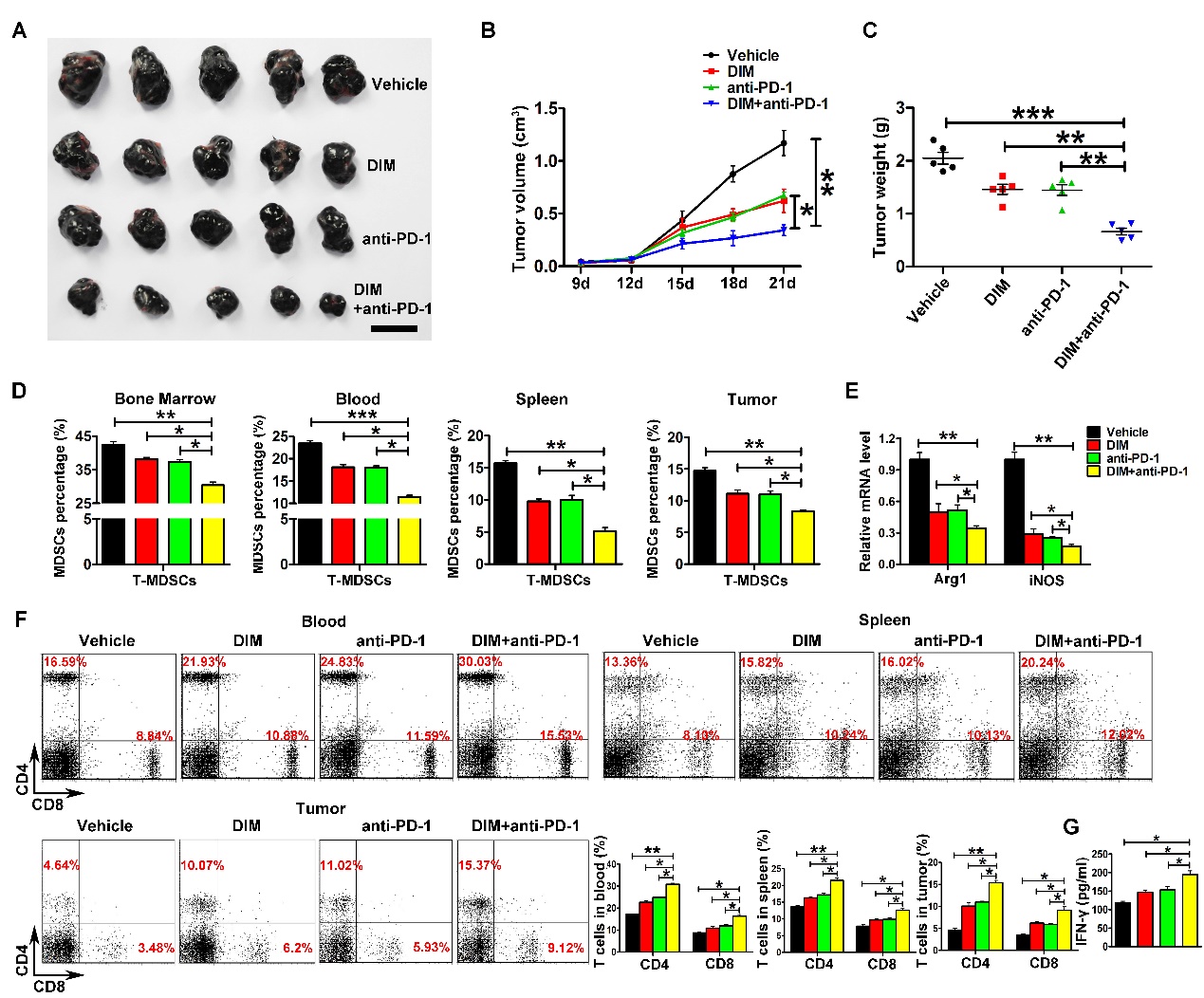


**Additional file 1: Fig S1 DIM treatment enhanced antitumor immune responses of anti-PD-1 in melanoma tumor-bearing mice.** Melanoma tumor-bearing mice were treated with 10 mg/kg DIM three times a week for two weeks from day 8 after tumor cell inoculation and intraperitoneally injected with anti-PD-1 mAb (0.25 mg/mouse) or isotype control antibody every two days from day 11 to 19 after tumor cell inoculation (n = 8). (A) Representative images of tumors, (B) tumor volume and (C) tumor weight were shown. (D) The percentage of total MDSCs from bone marrow, blood, spleen and tumor was examined by ﬂow cytometry. (E) Relative Arg1/iNOS mRNA levels in MDSCs from spleen of melanoma tumor-bearing mice after co-treated with DIM and anti-PD-1. (F) The percentage of CD4^+^ and CD8^+^ T cells from blood, spleen and tumor in melanoma tumor-bearing mice after co-treated with DIM and anti-PD-1. (G) IFN-γ level in tumor was measured by ELISA. Data are representative results from three independent experiments and the results are expressed as the mean ± SEM. **p* < 0.05, ** *p* < 0.01 and ****p* < 0.001. T-MDSC: total MDSCs; M-MDSC: monocytic MDSC; G-MDSC: granulocytic MDSC.


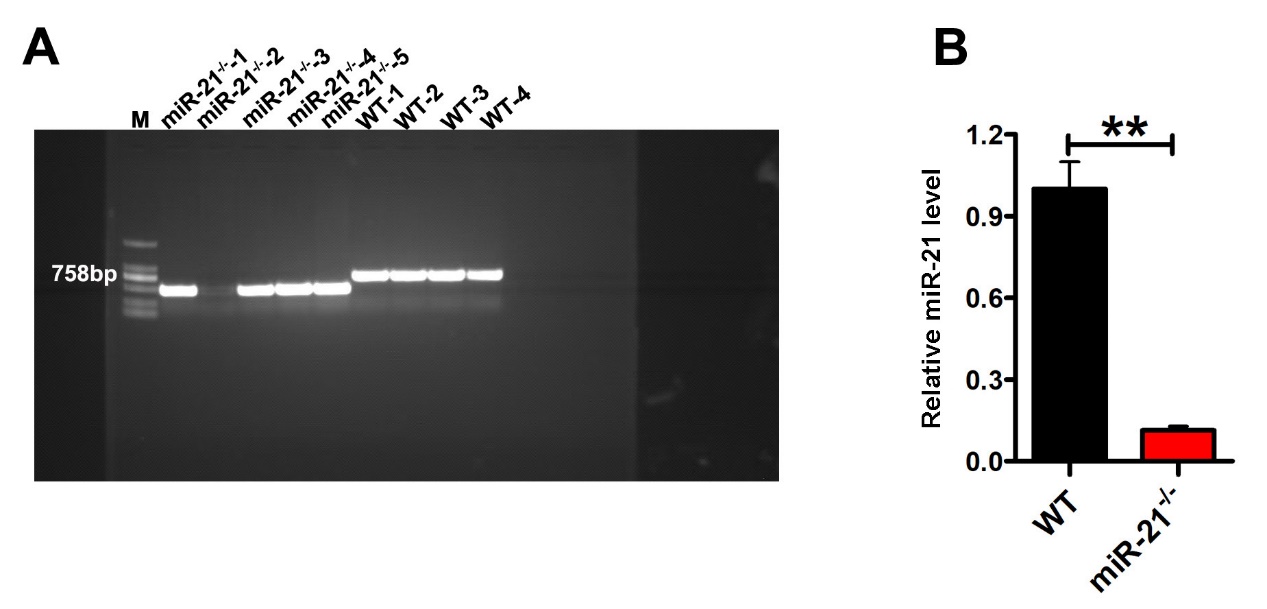


**Additional file 1: Fig S2 The characterization of *miR-21*^-/-^ mice.** (A) The genotyping analysis was performed by mouse tail PCR and agarose gel electrophoresis to confirm the deletion of miR-21. (B) Relative miR-21 level in MDSCs from the spleen of miR-21^-/-^ mice compared with WT mice. Data are representative results from three independent experiments and the results are expressed as the mean ± SEM. ***p* < 0.01.


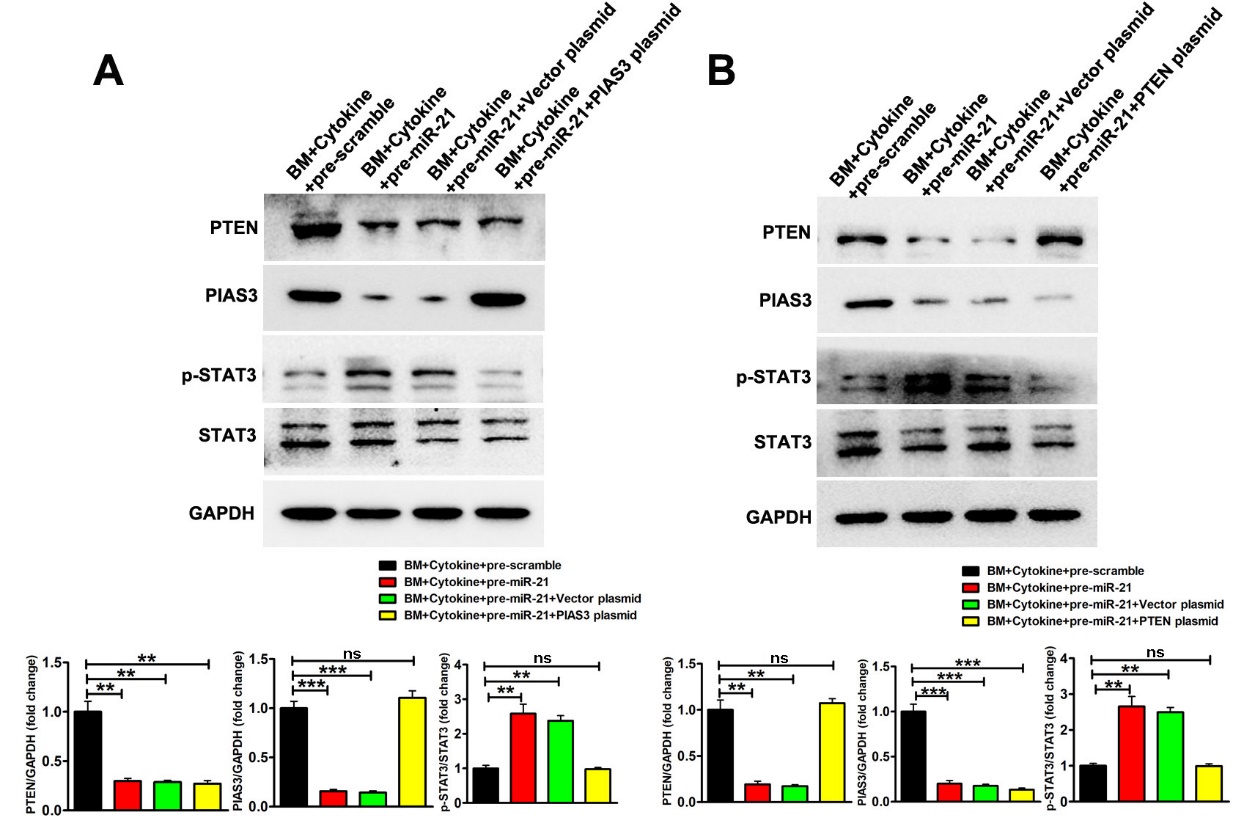


**Additional file 1: Fig S3 Overexpression of PIAS3 or PTEN partly abolished the effect of pre-miR-21 on STAT3 signaling pathway.** The BM-induced MDSCs (40 ng/ml GM-CSF and 40 ng/ml IL-6) were transfected with miR-21 precursors (pre-miR-21) or co-transfected with PIAS3 (A) or PTEN (B) plasmid and pre-miR-21 for 4 days. The protein levels of PTEN, PIAS3, STAT3 and p-STAT3 were examined by Western blot. Data are representative results from three independent experiments and the results are expressed as the mean ± SEM. ***p* < 0.01 and ****p* < 0.001. ns: no significance.


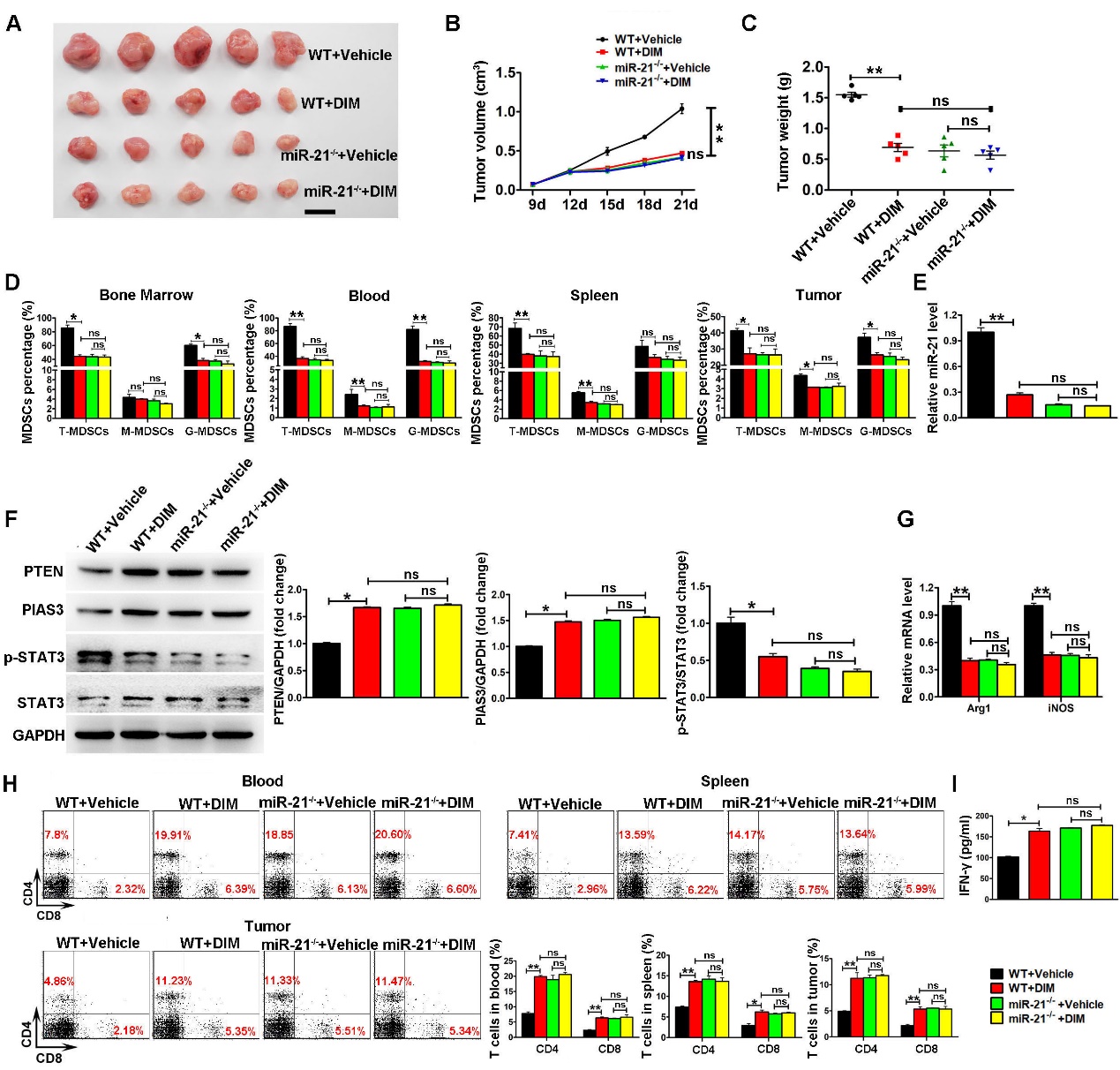
**Additional file 1: Fig S4 miR-21 deletion impaired the inhibitory effects of DIM on the expansion of MDSCs and tumor growth.** 4T1 tumor-bearing WT mice or *miR-21*^-/-^ mice were intraperitoneally injected with β-cyclodextrin or 10 mg/kg DIM three times a week for two weeks from day 8 after tumor cell inoculation (n = 8). (A) Representative images of tumors, (B) tumor volume and (C) tumor weight were shown. (D) The percentage of total MDSCs and their subsets from bone marrow, blood, spleen and tumor was examined by ﬂow cytometry. (E) Relative miR-21 level in MDSCs from the spleen of 4T1 tumor-bearing WT mice or *miR-21^-/-^* mice treated with DIM. (F) The protein levels of PTEN, PIAS3, STAT3 and p-STAT3 in splenic MDSCs of WT mice or *miR-21^-/-^* mice treated with DIM were examined by Western blot. (G) Relative mRNA levels of Arg1 and iNOS in MDSCs from spleen of 4T1 tumor-bearing WT mice or *miR-21*^-/-^ mice treated with DIM. (H) The percentage of CD4^+^ and CD8^+^ T cells from blood, spleen and tumor in DIM-treated tumor-bearing WT mice or *miR-21*^-/-^ mice. (I) IFN-γ level in tumor was measured by ELISA. Data are representative results from three independent experiments and the results are expressed as the mean ± SEM. **p* < 0.05, ***p* < 0.01 and ****p* < 0.001. ns: no significance. T-MDSC: total MDSCs; M-MDSC: monocytic MDSC; G-MDSC: granulocytic MDSC.
