# Supplementary material for: 3,3′-Diindolylmethane improves antitumor immune responses of PD-1 blockade via inhibiting myeloid-derived suppressor cells
Source: Chin Med. 2022 Jun 30;17:81. doi: 10.1186/s13020-022-00638-z (PMC9245307; doi:10.1186/s13020-022-00638-z)
Supplement: Supplementary file 2 — Additional file 2: Table S1. The primer sequences used in this study. Table S2. The antibodies used in this study. [file 13020_2022_638_MOESM2_ESM.docx]

**Additional file 2: Table S1-2.**

**Additional file 2: Table S1** The primer sequences used in this study

| Gene | Sequence (5’ to 3’) |
| --- | --- |
| *miR-21*^-/-^ mice genotyping  Forward | CAGAATTGCCCAGGCTTTTA |
| miR-21-/- mice genotyping Reverse | AATCCATGAGGCAAGGTGAC |
| miR-21 Forward | ACACTCCAGCTGGGTAGCTTATCAGACTGA |
| Reverse | CTCAACTGGTGTCGTGGAGTCGGCAATTCAGTTGAGTCAACATC |
| URP | TGGTGTCGTGGAGTCG |
| U6 Forward | CTCGCTTCGGCAGCACA |
| U6 Reverse | AACGCTTCACGAATTTGCGT |
| mus-PIAS3 Forward | GGACGTGTCCTGTGTGTGACAA |
| Reverse | ATCTCATCACAATCCGAACAGGAA |
| mus-Arg1 Forward | CTCCAAGCCAAAGTCCTTAGAG |
| Reverse | AGGAGCTGTCATTAGGGACATC |
| mus-iNOS Forward | CCAAGCCCTCACCTACTTCC |
| Reverse | CTCTGAGGGCTGACACAAGG |
| mus-Spray1 Forward | CATAGGTCAGATCGGGTCATC |
| Reverse | CAAGCAGGGTAGGCACAAA |
| mus-Spray2 Forward | CAGATCAGAGCCATCCGAAAC |
| Reverse | CAGACCCAAATCATCCTTGC |
| mus-PDCD4 Forward | GCTGCTCTGGATAAGGCTAC |
| Reverse | GTGATTGACAGGCTGTTGC |
| mus-PIK3R1 Forward | CTGGGAGAGAGCAGGCAAATTA |
| Reverse | CTATACGGCCCGCACTGTAA |
| mus-Tipe2 Forward | TCTCGCTCCCATCCAGTTCC |
| Reverse | TGTCCTAGTGCTGCCTCCAA |
| mus-btg2 Forward | GCGAGCAGAGACTCAAGGTT |
| Reverse | TAGCCAGAACCTTTGGATGG |
| mus-PTEN Forward | CGGCAGCATCAAATGTTTCAG |
| Reverse | AACTGGCAGGTAGAAGGCAACTC |
| mus-MEF2C Forward | ACTTGTGCAGAGGGATCACG |
| Reverse | TGCCTTTCTGCTTCTCCAGG |
| mus-cyclinD1 Forward | TCAAGTGTGACCCGGACTG |
| Reverse | CTCCTTCCTCTTTGCGGGTG |
| mus-β-actin Forward | GGTGTGATGGTGGGAATGGG |
| Reverse | ACGGTTGGCCTTAGGGTTCAG |

**Additional file 2: Table S2** The antibodies used in this study

| Antibody | Poly/monoclonal | Manufacturer | Dilution |
| --- | --- | --- | --- |
| P-STAT3 | Monoclonal | Cell Signaling Technology Inc. (#9145) | 1:2,000 |
| STAT3 | Monoclonal | Cell Signaling Technology Inc. (#8232) | 1:1,000 |
| PIAS3 | Polyclonal | Cell Signaling Technology Inc. (#4164) | 1:1,000  (WB) |
| PTEN | Monoclonal | Cell Signaling Technology Inc. (#9188) | 1:1,000  (WB) |
| PDCD4 | Monoclonal | Cell Signaling Technology Inc. (#9535) | 1:1,000  (WB) |
| HRP-conjugated goat anti-rabbit IgG | Monoclonal | Jackson ImmunoResearch (111-005-003) | 1:2,000 |
| HRP-conjugated anti-GAPDH | Monoclonal | KangChen Bio-tech Inc.(KG-5G5) | 1:10,000 |
